# “Making Peace” with Bodies and Sexual Selves: Changes during COVID-19 among Adults in the United States

**DOI:** 10.3390/ijerph182111063

**Published:** 2021-10-21

**Authors:** Jessamyn Bowling, Erin Basinger, Erika A. Montanaro

**Affiliations:** 1 Department of Public Health Sciences, University of North Carolina at Charlotte, Charlotte, NC 28223, USA; 2 Department of Communication Studies, University of North Carolina at Charlotte, Charlotte, NC 28223, USA; ebasinge@uncc.edu; 3 Department of Psychological Science, University of North Carolina at Charlotte, Charlotte, NC 28223, USA; emontana@uncc.edu

**Keywords:** body image, pandemic, COVID-19, body acceptance, self-image

## Abstract

The COVID-19 pandemic has negatively impacted the physical and mental health of many and has necessitated widespread societal shifts, including changes to work and family activities. These changes have impacted individuals’ identity, including their sexual self-image and body image, yet research on perceptions of these changes is missing. This study reports on quantitative and qualitative data from an electronic survey with adults in the United States (*N* = 326) to examine these perceptions. Body appreciation did not significantly differ between demographic groups. Themes emerging from the qualitative results included changes in general self-image (becoming more restricted or disempowered), changes in sexual self-image (deepening, becoming more sexy/sexual, or less sexy/sexual), and changes in body image (positive, negative, and neutral). Our findings point to positive, negative, and neutral effects on sexual self-image and body image, implying that nuanced approaches are needed to understand how identity has transformed as a result of the COVID-19 pandemic.

## 1. Introduction

COVID-19 has direct effects on people [1] e.g., difficulty breathing, fatigue, loss of taste or smell, nausea, gastrointestinal distress; as well as indirect psychological, emotional, and relational effects. Social distancing guidelines, work from home orders, and shelter-in-place ordinances, though protective of physical health, preclude ordinary face-to-face social interaction. Preliminary research indicates that COVID-19 may increase loneliness [2], stress [3], suicidality [4,5], and interpersonal violence [6,7].

One salient impact of COVID-19 is on people’s identity, or sense of self, given the isolation and disconnection that many have experienced. A few studies have specifically explored the links between COVID-19 and identity. Jaspal and Nerlich [8], for instance examined the relevance of social representations theory and identity process theory to the COVID-19 context. Other scholars have investigated how COVID-19 impacts identity in particular contexts, including teaching communities about COVID-19 prevention [9], moral injury in healthcare professionals [10], and discrimination and mental health [11]. Broadly, we know from the literature exploring the impact of cultural trauma that this type of trauma often leads to changes in core personal beliefs [12,13]. We argue that two core personal beliefs include our body image and concept of a sexual being, and that they have both been challenged as a result of the cultural trauma of the COVID-19 pandemic.

Our goal in this study was to investigate the influence of COVID-19 on two specific facets of identity: sexual self-image, or how one sees oneself as a sexual being, and body image. Indeed, body image has implications for sexual functioning [14] and vice versa [15]. We use the term “sexual self-image” to distinguish from some of the closely related concepts such as “sexual self-concept,” which can be defined synonymously with sexual self-image [16,17] e.g., “the feelings a person has about themselves as a sexual being”; or more often, include sexual self-efficacy, arousal, exploration, lack of anxiety, and sexual self-esteem [18]. Due to the limited research on sexual self-image, our rationale draws from these closely related concepts. In a multidimensional view of sexual health, sexual self-concept has been positioned as foundational for other domains [19,20]. Sexual self-concept has been linked to sexual behaviors [21,22], sexual functioning [23], and sexual satisfaction [24,25] and sexual self-esteem increased sexual self-efficacy [26]. Not only does sexual self-image impact sexual health outcomes, it also is affected by sexual health. Individuals with a sexually transmitted infection (specifically herpes or HPV) were more likely to have negative sexual self-concept [16].

From a qualitative, explorative standpoint, we allowed participants to determine what was salient for them about their sexual self-image rather than prescribed domains. Although most of the research related to the COVID-19 pandemic and sexuality focused on sexual behaviors, we sought to widen the scope of existing research by focusing on how people view themselves as whole sexual beings. Such a focus necessarily includes attention to individuals’ relationships with their bodies, as sexual health includes “physical, emotional, mental, and social wellbeing in relation to all aspects of sexuality and reproduction, not merely the absence of disease, dysfunction, or infirmity” [27]. Thus, our objective was to examine intersections among sexual self-image and body image.

### 1.1. COVID-19 and Identity

Theories of identity argue that identity formation is a fluid and dynamic process that occurs across the lifespan. Social identity theory, for example, suggests that identity is based largely on social comparison, as individuals identify with some people as ingroup members and differentiate themselves from others as outgroup members [28,29]. As social environments change, so does the negotiation of identity [30,31]. Similarly, the communication theory of identity contends that identity is multilayered, consisting of personal, enacted, relational, and communal components, all of which shift and develop over time [32,33]. Finally, identity theory claims that identity formation rests on the reciprocal relationship between social structures and the self, which is variant across the life course [34,35]. Common to all of these theories are the ideas that (a) identity formation is ongoing and (b) individuals forge their identities in connection with other people.

Identity theories point to major life transitions as catalysts for identity changes. These moments have been referred to as turning points, which mark important or salient events in the life course [36]. Strauss [37] defines turning points as “critical incidences [that] force a person to recognize that ‘I am not the same as I was, as I used to be’” (p. 149). In this study, we argue that COVID-19 is likely a transition that has influenced people’s sense of self, so our focus is on how the pandemic, conceptualized as a turning point, influences sexual identity and body image.

Though there are many factors that comprise identity (e.g., personal sense of self, social interactions, communal identity), we focus on sexual sense of self and body image in this study. These two components of identity are intricately related [38]. For example, negative messages from a sexual partner about one’s body decreased confidence, self-acceptance, and sexual empowerment [39]. Moreover, body image is related to sexual satisfaction [40] and arousal and orgasm [41]. Beyond their relationships to one another, sexual identity and body image are also important to overall health and wellbeing. For instance, women’s body satisfaction and sexual self-schemas were both related to life satisfaction and positive affect [42]. 

### 1.2. COVID-19 and Sexuality

Within the United States, the COVID-19 pandemic has affected sexual behaviors [43,44,45], relationships and intimacy [46,47], sexual drive [48], perceptions of sexual risk [49], and technology use in sexuality [50].

Relationships and intimacy have been challenged with the circumstances of the pandemic. Couples have been physically distant or confined together throughout the pandemic [46]. In addition, emotional distance or closeness has been influenced by widespread psychological distress or increased time spent with one another, among other factors [46,47]. Technology has been useful for some partners to maintain intimacy during social distancing, both through sexting (sharing sexual text or photos) as well as through video calls [46,50]. However, Banerjee and Rao [50] point out that solo sexuality has also been aided by technology use during the pandemic, with increases in masturbation and pornography use.

Sexual behaviors have been a focus of early COVID-19 research. Sexual drive and desire has often been reported as decreased, with some experiencing increases or deepening their understanding of their sexuality [48]. Some scholars reported overall decreases in sexual behavior [43] or noted that frequency of sexual intercourse was generally the same, on average [27]. Others offered more nuance in their reports. Coombe et al. [51], for instance, noted that sex with a dating partner or casual hook-up was reported less frequently, whereas sex with a spouse was more common during lockdown. In the same study, the authors reported an increase in masturbation and in solo use of sex toys [51], and multiple studies identified an increase in pornography consumption [27,52]. In another report, frequency of sexual behavior was dependent, in part, on the age of children in the home; those with children under five reported increased intimate behaviors, whereas those with children aged 6–12 years reported a decrease in the same behaviors [43]. Finally, some studies identified nuances based on sex or gender. For instance, nearly 40% of Jacob et al.’s [53] sample engaged in some sexual activity at least once per week, and this was particularly true for younger, employed men. Taken together, these studies illustrate that though sexual behavior has likely changed, pinpointing a pattern in those changes is challenging because sexual behavior is dependent on so many other factors.

One of those factors may be social isolation. Several scholars, for example, have argued that social isolation has a negative psychological impact, which in turn harms libido [54,55,56]. Offering empirical support for this hypothesis, Hensel et al. [43] found that depression and loneliness were related to reduced partnered sexual behaviors. Two other potential pathways are also concerned with psychological health, suggesting that depression affects sleep patterns, which can reduce desire for sexual activity [54] or that depression medication can decrease libido [54,57]. Relatedly, stress can have physiological effects on sexual functioning [27,57], and COVID-19 may decrease immune response, which alters sexual functioning [54]. A final pathway is related to risk behavior: people may not want to take on new sexual partners because of the risks associated with sex or a fear of contagion [27,55,58]. To date, these explanations are speculative, but they have received substantial attention in academic reviews and commentary related to COVID-19.

Other work on COVID-19 and sexual identity focuses on intimacy, particularly in established or long-term romantic couples. Scholars argued that intimacy could be strengthened for some couples because of increased time together, fewer social or family obligations, or less work burden, but they also cautioned that several factors could damage intimacy, including stress, lack of privacy, and increased chances for interpersonal conflict [57,59]. Similarly, Ibarra et al. [27] suggested that being together all the time could exacerbate conflict and weaken couples’ relational bonds, which in turn could harm sexual behavior. There is some preliminary empirical support for these arguments. For instance, only half of Arafat et al.’s [59] sample reported positive changes to their emotional bonding. Approximately one-third of participants in Luetke et al.’s [60] study noted that they had experienced COVID-19-related conflicts in their partnership, and conflict frequency was related to less frequent sexual behaviors, decreased experiences of orgasm, and feeling emotionally distant from one’s partner during a sexual event. Clearly, there is potential harm to romantic relationships because of the challenges of COVID-19.

### 1.3. COVID-19 and Body Image

Similar to the nascent literature focused on the relationship between COVID-19 and sexual identity, the emerging work on COVID-19 and body image hypothesizes a relationship between COVID-related stress and anxiety and increased rates of body dissatisfaction [61,62]. Recent empirical work has supported these hypotheses, e.g., [63,64,65]. For example, Swami et al. [63] sought to understand how stress and anxiety from the pandemic related to body image when controlling for other relevant psychological variables such as perceived stress, stressful life events, and trait anxiety. They found empirical support that COVID-19-related stress and anxiety were significantly associated with negative body image, and that the relationship between COVID-19-related stress and negative body image was more pronounced for women. Flaudias et al. [64] explored similar questions but focused on an undergraduate student population. They found that pandemic-related stress was related to a greater likelihood of binge eating and dietary restrictions, and that this relationship was especially true for participants who identified as female. Flaudias et al. [64] also highlighted that the pandemic may create body image and eating concerns for those already at-risk for problematic eating behaviors. Finally, Robertson et al. [65] provided a more nuanced picture in understanding perceived changes in body image in a UK population. The authors found that women, specifically young women, were more likely to report changes in their thoughts and behaviors related to their bodies, which broadly mirrors other literature focusing on the overall mental health impact of the COVID-19 pandemic [66,67]. Robertson et al. [65] also highlighted the association between changes in body image to reports of higher levels of psychological distress related to COVID-19. This may also be linked to changes in sexual identity and activity.

The pandemic and stay-at-home orders, in particular, have been stressors on individuals’ daily lives, interrupting routines, exercise habits, and accessibility to food [63,68]. Additionally, social media use has dramatically increased during this time [69], with a corresponding rise in content related to stigmatizing messages about weight gain [70,71] e.g., “Quarantine 15”; body shaming, and conflicting messages regarding the relationship between weight and COVID-19 risk [65]. Indeed, Flaudias et al. [64] found that increased exposure to COVID-19-related media was significantly associated with restrictive eating behavior, which, in turn, was associated with higher levels of body dissatisfaction. Clearly, the pandemic has impacted how individuals interact with their bodies, and preliminary evidence suggests it is often detrimental to psychological well-being.

### 1.4. The Current Study

In the current study, our goal is to add to the burgeoning literature on COVID-19, sexual behavior, and identity by focusing on how people have experienced changes to their sexual sense of self and body image during the pandemic. Given the evidence of impacts for both changes in sexual being and body image as a result of the pandemic, we examine them together to highlight nuances and interactions between these two domains. Whereas most prior work has focused narrowly on sexual behavior, we widened the lens in this study by asking people about their sexual sense of self more broadly, including not only behavior, but also their perceptions of their sexual selves, their ways of interacting with others, and their relationships with their own bodies. Accordingly, our study was guided by the following research question: How do people experience changes to the ways they think about themselves, including their sexual sense of self and body image, during COVID-19?

## 2. Materials and Methods

We conducted an online survey with open- and closed-ended questions with individuals in the United States over the age of 18. The larger study examined the effects of the COVID-19 pandemic on sexuality and relationships. This analysis focuses on changes to self-image and body image. Participants were recruited through snowball recruitment, health-related listserv announcements, social media postings in health-related groups (including COVID-19-specific groups) and population-related groups (to include underrepresented groups such as people with disabilities and LGBTQ individuals). Participants provided electronic consent and were entered into a drawing for an e-gift card upon completion of the survey. All procedures and protocols were approved by the authors’ institutional review board. Anonymous data files are available upon reasonable requests to the authors.

### 2.1. Participants

A total of 326 participants completed the survey. Participants varied in age (range 18–77); however, on average, participants were 30.6 years old (SD = 11.22). The majority of participants identified as women (*n* = 247, 76.0%), with smaller proportions of men (*n* = 73, 22.5%) and gender nonbinary and agender identifying individuals (*n* = 5, 1.5%). Participants overwhelmingly reported they identified as White (*n* = 246, 75.7%), with 6.5% (*n* = 21) who identified as Black, 6.5% (*n* = 21) as Hispanic/Latinx, 4.0% (*n* = 13) as Asian, 1.2% (*n* = 4) as American Indian, 0.3% (*n* = 1) as Middle Eastern, 5.5% (*n* = 18) as two or more identities, and 0.3% (*n* = 1) did not specify. Most participants identified as heterosexual (70.8%; *n* = 230), 3.7% (*n* = 12) as gay, 1.8% (*n* = 6) as lesbian, 12% (*n* = 39) as bisexual, 0.3% (*n* = 1) as pansexual, 2.5% (*n* = 8) as queer. 0.9% (*n* = 3) as asexual, 0.9% (*n* = 3) as other, and 7.1% (*n* = 23) as two or more identities. The majority of participants were in a committed relationship with one person (*n* = 210, 64.4%), whereas 20.2% (*n* = 66) of participants were single or not dating, 10.1% (*n* = 33) were dating casually, 1.2% (*n* = 4) were in a committed relationship with more than one person, 2.5% (*n* = 8) selected two or more statuses, and 1.5% (*n* = 5) reported other.

### 2.2. Procedures

#### 2.2.1. Measures

The overall survey took approximately 15 min to complete via Qualtrics. Relevant open-ended questions including “How has your sexual being changed since the COVID-19 pandemic began?”; “How have those changes influenced your larger sense of self, or how you see yourself?”; and “How have those changes influenced your sense of empowerment, or feeling capable and strong?”.

The 10-item Body Appreciation Scale-2 [72] (BAS-2); was used to evaluate participants’ acceptance and respect for their bodies. Participants indicated how often, on a 5-point Likert scale, 10 statements were true about them. Sample items included “I respect my body” and “I appreciate the different and unique characteristics of my body.” Responses ranged from 1 = Never to 5 = Always. Items were averaged to form a total score, with higher scores indicating higher levels of body appreciation (*M* = 3.59, *SD* = 0.82, α = 0.95). 

#### 2.2.2. Data Analysis

Bivariate analyses were conducted to explore potential demographic differences in body appreciation. Specifically, a correlation was conducted to determine if a relationship existed between age and body appreciation. A series of one-way ANOVAs were also conducted to determine if differences in body appreciation existed by gender, sexual orientation, or relationships status. Open-ended questions were coded in Dedoose [73]. We used inductive thematic analyses in which common ideas were grouped together to create themes [74]. All responses were coded by two coders trained in qualitative analyses. An initial codebook was created through preliminary analyses. After an initial coding of 30 participants, the codebook was augmented. The Dedoose “test” function confirmed coder interrater reliability; any codes with less than 0.8 Kappa were discussed and refined. 

## 3. Results

### 3.1. Quantitative Results

To explore a potential bivariate relationship between age and body appreciation, a correlation between the two variables was calculated. There was not a significant relationship between age and body appreciation, *r*(187) = 0.02, *p* = 0.84. A series of one-way ANOVAs were also calculated to determine if differences in body appreciation existed by gender, sexual orientation, or relationship status, respectively (see Table 1). Body appreciation did not differ significantly by gender (*F*(3,182) = 1.19, *p* = 0.32), sexual orientation (*F*(8,178) = 1.29, *p* = 0.25), or relationship status (*F*(5,181) = 0.86, *p* = 0.51).

### 3.2. Qualitative Results

#### 3.2.1. General Self-Image

Participants’ general self-image was more reserved (Table S1, 1a) or honed through doing things alone (Table S1, 1b,c). The isolation from others negatively affected some participants’ self-image, including their sense of empowerment (Table S1, 1d). Part of this related to individuals’ routines that were disrupted along with their sense of themselves as disciplined (Table S1, 1e). Relationships and sexuality that were limited due to the pandemic negatively affected participants’ overall self-image (Table S1, 1f,g). Some participants worried about the permanence of these changes in their self-image (Table S1, 1h).

#### 3.2.2. Sexual Self-Image

In relation to participants’ sexual self-image changes as a result of the pandemic, participants (a) deepened their understandings of themselves, (b) viewed themselves as more sexy or sexual, or (c) viewed themselves as less sexy or sexual. There were a few participants who described movement in both positive and negative ways, specifically in deepening understandings of self while at the same time having reduced opportunities for sexual engagement or feeling sexy (Table S1, 2a–c). Some participants were questioning their sexuality (mostly their sexual identity; Table S1, 2d) and learning to love themselves through their sexuality—including kinks (Table S1, 2e,f). One participant’s pride in their sexual identity off-set their insecurities about sexual inactivity (Table S1, 2g). Quarantine provided some participants the opportunity to re-examine their sexual self-image, to be more “loving and open” (Table S1, 2h) or to reframe their self-image of having a low sex drive (Table S1, 2i). The view of oneself as more sexual led some participants to more positive self-image overall (Table S1, 2j). Some participants contextualized their sexuality as just one component of their overall being (Table S1, 2k).

A large subtheme focused on participants viewing themselves as less sexy or sexual. Participants pointed to mental health difficulties, specifically depression, anxiety, or distraction, as one of the drivers of this change (Table S1, 2.1a–c). For others, changes in relationships with others was the key shift—including appearance to others (Table S1, 2.1d), practicing kinks (Table S1, 2.1e), and relationship partners (Table S1, 2c).

Participants drew linkages between how they viewed themselves as sexual and their gender. This was primarily true for participants identifying as cisgender women. A few participants seemingly equated being a (strong) woman with being sexual (Table S1, 2.2a,b).

#### 3.2.3. Body Image

Participants discussed positive changes in their body image related to partners—both in spending time together (Table S1, 3a) and in sex drive (Table S1, 3b). Losing weight also increased some participants’ sense of positive body image (Table S1, 3c).

Negative body image was part of some participants’ introspection during the pandemic. Participants described noticing their body image was based on looks (rather than feelings of being in the body; Table S1, 4a), the way the pandemic was affecting their eating disorder (Table S1, 4b), and attempting to make peace with their body (Table S1, 4c). A few participants noticed how age was affecting their perceptions, such as making them feel less attractive (Table S1, 4d).

Participants’ negative body image was linked to sexual desirability. Participants described feeling undesirable (Table S1, 4.1a) or less sexy (Table S1, 4.1b,c). For some, this reduction in sexual desirability was tied to self-esteem and self-worth (Table S1, 4.1d,e). Reductions in positive body image were tied to (potential) partners. With quarantining and reduced abilities to interact with others, some participants felt they were taking less care of themselves (Table S1, 4.1f). With less validation from partners, some participants struggled with navigating their insecurities (Table S1, 4.1g). Decreased desirability hindered some participants’ sexual interactions with partners (Table S1, 4.1h,i).

Many participants touched on body image in relation to exercise, both positive and negative. Participants’ abilities to exercise shifted during quarantine, which increased self-esteem for some (Table S1, 5a). The shift in part occurred because individuals had more time without a commute to work (Table S1, 5b). The pandemic also created opportunities for community building in relation to exercise, which facilitated some participants in working out (Table S1, 5c). However, some participants worked to navigate their exercise routines and eating in the upheaval of the pandemic (Table S1, 5d). On the negative side, some participants were not able to go to their regular gyms or exercise classes, which reduced their self-esteem (Table S1, 6a,b). This lack of physical activity reduced sex drive (Table S1, 6c). Participants ascribed lower amounts of activity to loss of self (Table S1, 6d,e).

For others, body image was shifting, but the effects were neutral. Without an “easy other” in their sexuality, participants described appreciating their body more holistically (Table S1, 7a). Similarly, another participant described their confidence as unchanging, and that confidence was the core of their sexiness (Table S1, 7b). For some older participants, the pandemic caused some to notice changes in their aging bodies (Table S1, 7c).

## 4. Discussion

Our goal in this investigation was to gain insight into the changes people experienced to both their sexual sense of self and their body image in the midst of the COVID-19 pandemic. Overall, participants reported positive, negative, and neutral changes in these areas. Importantly, our results suggest that body image and sexual sense of self are intricately tied to one another. For instance, positive body image was related to increased sex drive, whereas participants struggled with negative body image when they felt less sexually desirable. Those who fared the best seemed to be the ones who saw their body in a holistic way, integrating confidence with sexiness and their sense of their bodies. This set of findings points to the complexities of studying body image and sexuality—because people experience these parts of their identity in tandem, they are difficult to separate for the purposes of treatment or research. Indeed, scholars have noted links between body image and sexual sense of self in a variety of contexts, including disability [75] and chronic illness [76]. Our findings echo prior research that suggests body image and sexual identity co-vary.

The results of our study complement existing research about the pandemic and identity in two important ways. First, most prior research focused on changes to patterns of sexual behavior specifically; we widened the lens to focus on sexual identity more broadly. Certainly, our participants noted changes to their sex drive, the frequency of their sexual engagement with others, and their sexual functioning, in line with existing work on COVID-19 (Hensel et al., 2020; Li et al., 2020; Sansone et al., 2020). However, participants also contextualized those ideas in light of larger changes to their sense of self—including feelings of self-worth, their understanding of themselves, evolving ideas about their sexuality, and mental health struggles. Second, our findings offer an explicit link between sexual identity and body image, in contrast to existing work that examines one or the other. Thus, our work implies that body image may be another potential pathway linking COVID-19 with sexual behavior, in addition to those that have already been proposed (e.g., psychological impact, stress, risk; Abbas et al., 2020; Ibarra et al., 2020; Gaspari et al., 2020).

### 4.1. General Self-Image

In terms of general self-image, some participants reported concerns about the permanence of disempowerment or restriction of themselves. In addition to general concerns about COVID-19 transmission and societal effects (including employment and financial concerns, as well as social relationships), individuals may be navigating worries about their identities. Some participants contextualize their different identities as only one part of themselves, which gave them stability in their general sense of self and/or sexual self-image. These findings reaffirm a rich history in psychology that points to the importance of multiple selves or identities [77,78]. Specifically, having a number of identities that can be relied upon when one or many are being threatened has been linked to higher psychological well-being, and this appears to be especially true in moments when identities are being shifted or integrated [79]. This finding suggests that the salience and importance of an identity or self-image to an individual must be considered in order to create effective prevention or intervention messages.

Participants reporting isolation or lack of social interaction often referenced negative effects on their sense of self, whereas individuals who deepened their self-awareness reported more positive effects. This may point to opportunities for cognitive reframing. Indeed, the post-traumatic growth literature points to the relationship between traumatic events (e.g., the COVID-19 pandemic) and identity, suggesting that trauma such as the pandemic can serve as a lens through which to guide identity development [80,81]. Our findings highlight the ways in which mental health practitioners and interventionists may approach the reported shifts in sexual self-image and body image due to the pandemic.

### 4.2. Sexual Self-Image

Individuals in our study reported changes in how they see themselves as sexual beings. The lasting effects of these changes post-pandemic are unknown, and research may be needed to follow-up on the continued shifts or support needs for these changes. The descriptions of decreases in sexual self-image, or seeing oneself as a less sexual person, may cause psychological distress. Mental health practitioners may provide support during and post-pandemic for potential concerns.

In the case of negative effects on sexual self-image, many participants pointed to connections with mental health symptoms (e.g., depression). This further highlights the overlapping and intersecting relationship between sexual self-image and mental health outcomes (Abbas et al., 2020; Gaspari et al., 2020; Sansone et al., 2020; Hensel, 2020). Societal awareness of the mental health impact of the pandemic is increasing; however, the influence on individuals’ sex lives and identity are often overlooked and this may negatively influence psychological wellbeing. Similarly, some participants discussed their struggles with eating disorders and exercise behaviors as routines, social interactions, and access to food and exercise regimens changed. Research has long emphasized a relationship between trauma and eating disorders, e.g., [82], and our results suggest that the pandemic may be a source of trauma that compounds disordered eating behaviors. Taken together, these findings highlight an intricate relationship between sexual identity, body image, and mental health that clinicians should attend to in their patients.

In connecting our exploratory results of sexual self-image to established literature on sexual self-concept, we find many linkages. Within Deutch, Hoffman, and Wilcox’s [18] conceptual model, we see evidence of sexual behavior and sexual attractiveness from their domain of sexual self-esteem and exploration as well as anxiety (though not only restricted to sexual situations but regarding sexuality broadly). However, we do not see sexual conduct (feelings of adequacy about one’s behavior in a sexual situation) or sexual self-efficacy. This is likely because other dominant themes such as attractiveness or arousal were more salient and our measure using short-form questions did not explore these further.

### 4.3. Body Image

Our results also highlight the fragility of people’s relationships with their body. Many participants reported changes in the way they felt about their bodies during the pandemic. Though some of those changes were positive, most positive changes were driven by external factors including time spent with a partner or weight loss. The former encourages positive body image based on feedback from a partner, whereas the latter relies on weight-normative attitudes that stress weight loss and conformity to socially enforced body norms (Tylka et al., 2014). Similarly, some participants reported reduced feelings of self-esteem when they were unable to exercise or, in contrast, increased self-worth because they had more time to exercise. Such dynamics showcase how easily people’s relationships with their bodies can change based on factors outside their control. Overall, this set of findings highlights the value of weight-inclusive approaches to health, such as Health at Every Size [83] and intuitive eating [84], given that the assumptions of these programs stress an internally guided relationship with one’s body.

Although we did not find significant differences in body appreciation scores, we did observe trends in the direction of lower scores among gender minority (nonbinary and agender) individuals and those who are single compared to those in relationships. We may not have had sufficient statistical power to detect significant differences among these groups. This trend comports with previous research that finds higher rates of body dissatisfaction among trans and nonbinary individuals compared to cisgender individuals [85,86]. Research in cisgender populations has uncovered differences among men and women, generally reporting lower body appreciation in women compared to men [87]. Our findings suggest that the COVID-19 pandemic may be negatively impacting body image in different ways for men and women. Specifically, women were more likely in open-ended responses to describe their sexual self-image as tied to their gender. Given the widespread nature of women’s sexual objectification [88], social changes in the pandemic may be disrupting the ways some women are viewing themselves. In our sample, men also reflected on negative body image, though not in context of partnerships or their gender but rather mostly just dissatisfaction with their bodies. Men’s negative body image comments coincide with literature pointing to men’s concerns about thinness, leanness, and muscularity at lower rates than women but nonetheless present [89,90,91,92].

Some participants described validation and affirmation from others in assisting them in their body and sexual self-image. Single participants may particularly struggle with lacking this external input.

### 4.4. Strengths and Limitations

We conducted this study relatively early in the pandemic, and follow-up studies may examine how these trends shift throughout the pandemic and when people are able to return to relative normalcy. Moreover, our sample was made up of predominantly White and cisgender women, and we were unable to examine how body and sexual self-image may have been affected for Black, Indigenous, and People of Color. Though we did observe some diversity in our sample in terms of sexual identity, research that focuses more centrally on the experiences of marginalized communities would be valuable in nuance to our results.

## 5. Conclusions

The COVID-19 pandemic has challenged people’s sense of self in a variety of ways as they navigate social distancing guidelines, work-from-home orders, and changing relationship dynamics. In this study, we focused on two facets of identity, sexual identity and body image, and the ways that people have experienced changes in those areas in the midst of the pandemic. Participants noted positive, negative, and neutral changes to their sexual self-image and body image and note how the two are intricately related to one another. Further, we see evidence of the pandemic’s effects across a wide range of aspects within sexual self-image. Our results highlight the importance of considering sexual sense of self and body image in tandem. Given the shifts in sexual self-image, mental and sexual health practitioners may need to focus on supporting individuals in making sense of themselves during and post-pandemic.

## Figures and Tables

**Table 1:** Body appreciation scores by demographic variables.

	Mean (SD)	Test Statistic
**Gender**		*F*(3,182) = 1.19, *p* = 0.32
Man	3.68 (0.77)	
Woman	3.58 (0.83)	
Nonbinary	2.80 (0.78)	
Agender	3.20 (0.00)	
**Sexual Orientation**		*F*(8,178) = 1.29, *p* = 0.25
Heterosexual/Straight	3.67 (0.80)	
Gay	3.09 (0.33)	
Lesbian	3.60 (1.64)	
Bisexual	3.40 (0.87)	
Pansexual	4.50 (0.00)	
Queer	3.96 (0.66)	
Asexual	3.63 (0.60)	
Other	3.63 (1.19)	
2+ Identities	3.21 (0.81)	
**Relationship Status**		*F*(5,181) = 0.86, *p* = 0.51
Single	3.38 (0.91)	
Dating casually	3.81 (0.67)	
In a committed relationship with one person	3.62 (0.82)	
In a committed relationship with more than one person	4.00 (0.44)	
Other	3.80 (0.14)	
2+ statuses	3.58 (0.49)	

## Data Availability

Data is available upon reasonable request to the authors.

## Supplementary Materials

The following are available online at https://www.mdpi.com/article/10.3390/ijerph182111063/s1, Table S1: Example quotes with participant demographic information (age in years, gender, sexual identity, race/ethnicity, relationship status).
