# Supplementary material for: “Making Peace” with Bodies and Sexual Selves: Changes during COVID-19 among Adults in the United States
Source: Int J Environ Res Public Health. 2021 Oct 21;18(21):11063. doi: 10.3390/ijerph182111063 (PMC8583655; doi:10.3390/ijerph182111063)
Supplement: Supplementary file 1 [file ijerph-18-11063-s001.zip › ijerph-1380755(1).pdf]

Table S1: Example quotes with participant demographic information (age in years, gender, sexual identity, race/ethnicity, relationship status)

**1. General self-image**	a) I am more reserved *(21; Cisgender woman; Heterosexual; Black/African American; Dating casually)*
	b) I feel more connected to myself since I'm taking time to do things alone…and feel as if I learned more about the person I am *(21; Cisgender woman; Heterosexual; White; In a committed relationship with one person)*
	c) Has not changed but see people less frequently (relationship more isolated) so awareness is more of self and in family structure. *(39; Cisgender woman; Queer; Black/African American; In a committed relationship with one person)*
	d) Being in quarantine has taken a huge hit to my empowerment and self esteem/mental health. *(21; Cisgender woman; Heterosexual; White; Dating casually)*
	e) This has affected my mood by interfering with my routine which is important for my sense of self with regard to discipline and structure. *(33; Cisgender man; Gay; White; In a committed relationship with one person)*
	f) I saw myself as someone who was in an exciting relationship, and now I am feeling more boring and lame because of this. *(29; Cisgender woman; Heterosexual; White; In a committed relationship with one person)*
	g) I view myself in a negative light because I am unable to lead a healthy sex life without barriers. *(24; Cisgender woman; Heterosexual; Hispanic/Latino/Spanish; In a committed relationship with one person)*
	h) I feel weak and worry I'll never get my old self back. I also feel embarrassed. *(25; Cisgender woman; Heterosexual; White; In a committed relationship with one person)*
**2. Sexual self-image**	a) My libido has decreased, my attraction to my roommate has increased, my desire for other sexual partners has increased, my desire for power exchange has increased, I'm flipping between feeling both less secure in my body and more certain of being sexy- they both happen and it fluctuates. Having less sex, but that's partly due to pain that can't be treated during the lockdown. *(31; Cisgender woman; Bisexual, Pansexual, Queer; White; In a committed relationship with more than one person)*
	b) More empowered/strong because I don't feel needy about sex or worry about rejection because I don't desire it as much. But I also feel less sexy, which feels less empowering too. I can't say which feeling is stronger. *(26; Cisgender woman; Heterosexual; White; In a committed relationship with one person)*
	c) My partner and I were able to see each other only a couple times over the last 2 months. During those times I was on my period, which always makes [sex] less satisfying for me, and makes me see myself as a less sexual being. I typically have trouble seeing myself as a sexual being, even though I masturbate and have sex regularly. However, I am reading *Come as You Are* during my quarantine, which has increased my self-compassion around body and sex, and changed my understanding of sex…It is certainly empowering to practice loving my body - seeing its positive attributes. and to practice self-compassion around movement, food, and sex helps

decrease negative self-talk in other areas as well. *(24; Cisgender woman; Bisexual; White; In a committed relationship with one person)*

d) I'm questioning my sexuality more. *(22; Cisgender woman; Other sexual identity; Black/African American; Single, not dating)*

e) I just love myself more. I respect my sexual identity more having had time to work on some of my trauma from being closeted and bisexual my whole life. *(28; Cisgender woman; Bisexual, Pansexual, Queer; White; Other)*

f) I've become more accepting of myself and my kinks. *(35; Cisgender man; Heterosexual; White; In a committed relationship with one person)*

g) Sometimes I may feel that I am sexually underactive for my age compared to my female and male peers…. Yes, the changes have made me feel insecure about my sexuality sometimes. Being bisexual has made me feel stronger because relationships with men may not feel as equal compared to being with a woman. *(22; Cisgender woman; Bisexual; Black/African American; In a committed relationship with one person)*

h) By being 'forced' to quarantine with my partner, I have had more opportunities to face my social anxiety, insecurities and mixed feelings about sex. My sexual being has become more loving and open! *(22; Cisgender woman; Bisexual; White; In a committed relationship with one person)*

i) I feel strong, I feel more independent. I always thought I had a low sex drive and this change of pace has been a nice relief from the labeling I put on myself. (*26; Cisgender woman; Heterosexual; White; In a committed relationship with one person)*

j) I have been seeing myself more and more like a super sexual being. I find that I feel braver and like myself a lot more. *(35; Cisgender woman; Bisexual; White; In a committed relationship with one person)*

k) I feel more complete and settled. My sexuality is in all parts of me - not just A part of me. *(43; Cisgender woman; Heterosexual; White; In a committed relationship with one person)*

2.1 Less sexual person

a) Makes me feel worse about myself to not be interested in anything sexual. Depression lowers sex drive. *(28; Cisgender woman; Pansexual; White; In a committed relationship with one person)*

b) This happens to me when I go through anxious episodes. So when my libido isn't what it usually is, it is frustrating because I really enjoy sex with my partner when I am not anxious/stressed. Sometimes I get insecure and feel that I am not sexual enough to sustain my relationship. *(26; Cisgender woman; Heterosexual; White; In a committed relationship with one person)*

c) Too distracted and off normal schedule to think about sexual activity, feel less sexual, like it is not a priority. *(40; Cisgender woman; Heterosexual; White; In a committed relationship with one person)*

d) I perceive myself less sexual because I don't get ready like I would if I was going out and about. *(28; Cisgender woman; Heterosexual; White; In a committed relationship with one person)*

e) COVID19 has seriously interrupted my sexual being. I was on a high in Dec/Jan/Feb. And had started going to kink events, started seeing more casual

	partners, entered a D/s relationship with a new partner. My confidence and sexuality has certainly been the biggest challenge and disappointment since this started… There has been a significant drop in my sense of self. It was very difficult to be plummeted from the most confident and fulfilled I have been in a long time to being isolated from my Dom and the kink community that led me to that fulfillment *(27; Non-binary; Queer; White; In a committed relationship with more than one person)*
2.2 Sexual person related to gender	a) My sexuality generally makes me feel strong, so lacking desire has certainly influenced how empowered I feel as a woman. *(36; Cisgender woman; Lesbian; White; In a committed relationship with one person)*
	b) I've never been confident as a sexual being/woman and that remains. *(28; Cisgender woman; Heterosexual; White; In a committed relationship with one person)*
**3. Body Image: Positive**	a) Recently, when we have sex is because we're both just very happy to be together and it makes me feel more attractive *(27; Cisgender woman; Heterosexual; Hispanic/Latino/Spanish; White; In a committed relationship with one person)*
	b) My low sex drive typically makes me view myself as a fairly non-sexual being, so the small increase in sexual desire the past 3 months has resulted in me reconsidering that. I actually feel more comfortable in my body these days than I have for a while and more confident in pursuing sex with my partner when I'm in the mood for it. In some ways, less stress from work has resulted in me feeling more sexy and more positive about being a "sexual being." *(27; Cisgender woman; Heterosexual; White; In a committed relationship with one person)*
	c) Since I have lost weight, I feel more attractive. *(32; Cisgender woman; Heterosexual; White; In a committed relationship with one person)*
**4. Body Image: Negative**	a) I no longer feel like the best version of myself and I have realized how much importance I put on my weight especially in the way I look more than how I feel *(28; Cisgender woman; Bisexual; White; In a committed relationship with one person)*
	b) I also struggle with an eating disorder [ED] so I don't feel sexy right now because I am not pleased with my body. The ED usually gets worse in situations of high stress/anxiety- like COVID19 *(26; Cisgender woman; Heterosexual; White; In a committed relationship with one person)*
	c) Feel challenged to make peace with my body *(26; Cisgender woman; Queer; White; Single, not dating)*
	d) I feel older and not as attractive. Clothes are not fitting as well and I actually like staying in sweat pants and loose fitting clothing more. It's a struggle to put on real clothes, comb my hair and go out to the grocery store. Also wearing a mask, I have given up trying to be sure the straps don't mess up my hair. The grey roots are growing more and more and I'm not really sure what to think about that. *(65; Cisgender woman; Heterosexual; White; In a committed relationship with one person)*
4.1 Negative body image related to sexual desirability	a) I feel gross and undesirable all the time. *(29; Cisgender woman; Heterosexual; White; In a committed relationship with one person)*
	b) The added belly fat has decreased my self perception as "sexy" *(32; Cisgender man; Heterosexual; White; In a committed relationship with one person)*

	c) I have gained weight over the last 3 months, and this has led to some body perception and ultimately sexual issues…I do not see myself as sexy or hot. *(21; Cisgender man; Gay; White; In a committed relationship with one person)*
	d) My libido is about the same but I feel much less desirable… It has lowered my self esteem and has made me more self conscious *(28; Cisgender woman; Bisexual; White; In a committed relationship with one person)*
	e) The biggest thing that has changed in the last three months has been how I perceive myself. Before the lockdown, I could find some comfort in my body, but since being home, my own sense of self has decreased and I do not feel sexy or worthwhile. *(23; Cisgender woman; Bisexual; Hispanic/Latino/Spanish, White; In a committed relationship with one person)*
	f) Feeling a bit powerless overall, and feeling a bit more negative about my body overall, don't feel like I'm taking care of myself as I used to. Miss the ability to go out, get dressed up and feel "sexy" *(34; Cisgender woman; Heterosexual; White; In a committed relationship with one person)*
	g) I think that validation from sexual and romantic partners supplements my self esteem somewhat and helps me to feel good about myself when I am low, and now with less of that I find it more difficult to face my physical and sexual insecurities on my own. It's also somewhat lonely. *(20; Cisgender woman; Heterosexual; American Indian/Alaska Native; Dating casually)*
	h) I feel lazier and bigger and not as comfortable with him [my partner]. *(28; Cisgender woman; Heterosexual; White; Dating casually)*
	i) I feel like my confidence and comfort with my sexuality has decreased in the past two months and that it is mostly attributed to an increasing struggle with body image. In my relationship, I am generally the initiator of sexual interaction and I am feeling more timid and less willing to suggest/initiate with my partner which in turn increases anxiety around body image and my perception of being attractive/desirable to my partner. *(37; Cisgender woman; Heterosexual; White; In a committed relationship with one person)*
**5. Body image related to exercise: Positive**	a) My perception of myself sexually has changed. For example I've felt a boost of self confidence from working out during quarantine. *(22; Cisgender woman; Heterosexual; Black/African American; In a committed relationship with one person)*
	b) Working from home and not traveling to work has given me 2 hours back a day so I am exercising, less stressed and sleeping better so feeling positive about me thus probably translate into more attractive. *(58; Cisgender woman; Heterosexual; White; Single, not dating)*
	c) Boxing has definitely helped. My boxing gym is all women, and that community has only gotten stronger during quarantine as we all come together to support each other, not just in fitness. …It's really surprised me what I'm capable of. If I can't punch the coronavirus, I can at least be a badass. *(30; Cisgender woman; Bisexual; White; In a committed relationship with one person)*
	d) I think throughout my time being stuck at home my self-confidence has fluctuated. At first, I was self-conscious because I was working out less and eating more but I have started doing yoga regularly and feel much better. *(21; Cisgender woman; Heterosexual; White; In a committed relationship with one person)*

**6. Body image related to exercise: Negative**	a) Without my yoga class, it has been more difficult to look at my full body reflection (face is fine and easy). I continue to struggle with body image and self acceptance that I struggled with before quarantine. I continue to struggle with negative thoughts and constantly try to counter those thoughts. *(30; Cisgender woman; Bisexual, Pansexual, Queer, Asexual; White; In a committed relationship with one person)*<br><br>b) Desire is much less. Gaining quarantine weight and feeling worse about my self-esteem. Not have as much sex as before…Yes, it has negatively affected by larger sense of self… It has been detrimental to empowerment or feeling capable and strong. I also can't go to the gym anymore so in a physical sense, I have also lost strength and muscle mass. *(29; Cisgender woman; Heterosexual; Asian; In a committed relationship with one person)*<br><br>c) I have more time and energy for sexual activity. "Normally" my partner and I wake up very early and are often too tired. Now that we're both home we have more time. However, I don't always feel "sexy" as I'm more sedentary than previously. *(34; Cisgender woman; Heterosexual; Hispanic/Latino/Spanish, White; In a committed relationship with one person)*<br><br>d) My increased sex drive hasn't changed how I see myself, but my increased weight from the less activity and more eating has given my sense of self a hit. *(33; Cisgender woman; Heterosexual; White; In a committed relationship with one person)*<br><br>e) A bit less confident due to not working out as much. … Less confident= less libido…I gain self worth through more channels than just my self appearance, but it still is impacting my overall image. *(26; Cisgender woman; Lesbian; White; In a committed relationship with one person)*
**7. Neutral body image**	a) [My sexual being] no longer includes an easy "other,'" so it's a very self-centered sexuality. That's not necessarily difficult to navigate, but it requires a change, and that means stuff like appreciating your body, feeling whole in your body. It's a process too. *(22; Cisgender woman; Bisexual; White; Dating casually)*<br><br>b) I think that I've always been comfortable with my sexuality and seen myself as a wanted woman. Even though my body has changed, the essence of who I am hasn't. I think that's what makes me sexy- the confidence I have in who I am as a being. *(42; Cisgender woman; Heterosexual; American Indian/Alaska Native, Black/African American, White; In a committed relationship with one person)*<br><br>c) It [the pandemic] has made me realize that I am aging and my body is changing. *(28; Cisgender woman; Heterosexual, Queer; White; In a committed relationship with one person, Other)*
